# Community-based delivery of intermittent preventive treatment of malaria in pregnancy in Burkina Faso: a qualitative study

**DOI:** 10.1186/s12936-021-03814-y

**Published:** 2021-06-23

**Authors:** Danielle Burke, Justin Tiendrebeogo, Courtney Emerson, Susan Youll, Julie Gutman, Ousmane Badolo, Yacouba Savadogo, Kristen Vibbert, Katherine Wolf, William Brieger

**Affiliations:** 1 Jhpiego, Baltimore, MD USA; 2 Jhpiego, Ouagadougou, Burkina Faso; 3 US President’s Malaria Initiative, Malaria Branch, Division of Parasitic Diseases and Malaria, Center for Global Health, US Centers for Disease Control and Prevention, Atlanta, USA; 4 US President’s Malaria Initiative, US Agency for International Development, Washington, D.C. USA; 5 National Malaria Control Programme, Ministry of Health, Ouagadougou, Burkina Faso; 6 The Johns Hopkins Bloomberg School of Public Health, Johns Hopkins University, Baltimore, MD USA

**Keywords:** Malaria, Pregnancy, Intermittent preventive treatment, Community healthcare workers

## Abstract

**Background:**

Burkina Faso is among ten countries with the highest rates of malaria cases and deaths in the world. Delivery and coverage of intermittent preventive treatment of malaria in pregnancy (IPTp) is insufficient in Burkina Faso; In a 2016 survey, only 22% of eligible women had received their third dose of IPTp. It is also an extremely rural country and one with an established cadre of community healthcare workers (CHWs). To better meet the needs of pregnant women, an enhanced programme was established to facilitate distribution of IPTp at the community level by CHWs.

**Methods:**

In order to assess the perceptions of CHWs and facility healthcare workers (HCWs) involved in this programme rollout, semi-structured interviews were conducted at three high malaria burden health districts in Burkina Faso. Interviews were conducted at baseline with 104 CHWs and 35 HCWs prior to the introduction of community based IPTp (c-IPTp) to assess capacity and any areas of concern. At endline, interviews were conducted with 29 CHWs and 21 HCWs to identify key facilitators and suggestions for further implementation of the c-IPTp programme.

**Results:**

CHWs reported feeling capable of supporting c-IPTp delivery and facilitating linkage to antenatal care (ANC). They noted that the opportunity for enhanced training and close and ongoing connections with facility HCWs and supportive supervision were imperative. Both CHWs and HCWs perceived this approach as acceptable to community members and noted the importance of close community engagement, monthly meetings between CHWs and facility HCWs, and maintaining regular supplies of sulfadoxine–pyrimethamine (SP). Those interviewed noted that it was beneficial to have the involvement of both female and male CHWs.

**Conclusions:**

Community-based delivery of IPTp was feasible and acceptable to both facility HCWs and CHWs. This approach has the potential to strengthen delivery and uptake of IPTp and ANC both in Burkina Faso and across the region.

## Background

Malaria remains a major public health problem worldwide, especially in sub-Saharan Africa with children under 5 years old and pregnant women among the most vulnerable [1]. The World Health Organization (WHO) recommends at least three doses of intermittent preventive treatment of malaria in pregnancy (IPTp) with sulfadoxine–pyrimethamine (SP) at least 1 month apart, beginning as early as possible from the start of the second trimester in order to reduce pre-term deliveries, low birth weight, and neonatal deaths [2, 3]. Results from the 2014 Burkina Faso Malaria Indicator Survey (MIS) showed that 68% of recently pregnant women received the first dose of IPTp (IPTp1), 48% received IPTp2, and only 22% received IPTp3 [4].

Low uptake of IPTp has been a common phenomenon across many high-burden malaria endemic countries [1, 5, 6]. It is normally delivered during the course of ANC visits at a healthcare facility. One approach that has been considered in response is delivery of IPTp by community healthcare workers (CHWs). Delivery of malaria services by CHWs is well established for delivery of diagnosis and treatment in children under 5 years of age [7–10] and in maternal and infant healthcare more broadly [11–13]. Community-based delivery has specifically been shown to improve IPTp uptake among pregnant women [14, 15] including in a previous study of female CHWs based in Burkina Faso [16] as well as when CHWs were deployed to conduct active malaria testing among pregnant women [17]. The intensified delivery of IPTp through an existing CHW network has the potential to boost the percentage of women reached for the recommended number of IPTp doses and enhance ANC attendance through CHW referrals.

Burkina Faso’s existing health system includes CHWs, known locally as *Agents Santé de Base Communautaire* (ASBC), who are trained and supervised by facility-level health staff. The CHW approach has been operational since at least 2008. Through the national CHW and community health programme, each village in the country is expected to select two CHWs who have completed primary school; the CHWs are paid CFA 20,000 (approximately USD 35) per month. The training manual for CHWs covers reproductive and maternal health, infectious diseases, including malaria, immunization, hygiene, and nutrition [18]. Most of the work in practice is health promotion and education, since the CHWs are yet to be adequately supplied with drugs and materials. Although CHWs have been taught to provide appropriate malaria treatment, malaria drugs are only available in a few pilot communities. The normal duties of a CHW include visiting women, educating them about various health issues, and referring them to the local health facility when necessary. Providing IPTp was not among the duties performed by CHWs [19] prior to the study.

To address the challenge of low IPTp uptake, the Burkina Faso government, with support from the U.S. President’s Malaria Initiative (PMI), conducted a cluster-randomized trial to determine the effect of community based IPTp (c-IPT) distribution on IPTp uptake and ANC coverage [20]. The intervention involved training existing CHWs to implement community sensitization activities around antenatal care (ANC) and malaria in pregnancy, and to refer pregnant women to ANC for their first dose of IPTp. Based on the WHO recommendation to initiate IPTp at the beginning of the second trimester, CHWs were taught by district-level staff to bring women to ANC for IPTp1 so that trained HCWs could accurately estimate gestational age. CHWs provided subsequent monthly SP doses. In addition, for villages where there was not an existing female CHW, the team recruited a female to work in the CHW role. This trial found an increase in median doses of IPTp among pregnant women in communities with this enhanced programme. At baseline, women received a median of 2.1 doses; by endline, women received a median of 1.8 doses in the control group and 2.8 doses in the intervention group (p-value < 0.0001). The study also found a non-statistically significant increase in the proportion of women attending four ANC visits in the intervention compared to the control group (difference in differences (DiD) = 12.6%, p-value = 0.16). Despite some successes in introducing c-IPTp, more information is needed about the perspectives of facility HCWs and CHWs regarding its implementation. Thus, as a sub-component of the study, HCWs’ and CHWs’ perspectives were assessed for feedback on roll-out of the community-based IPTp programme.

## Methods

To assess experiences and perceptions of facility HCWs and CHWs regarding implementation of the new c-IPTp programme, a qualitative study was conducted through in-depth interviews with facility based HCWs and CHWs using structured interview guides. Baseline interviews were conducted in May 2017 and endline interviews in August 2018 following programme introduction. The interviews were conducted in intervention areas which received the introduction of c-IPTp. The study was implemented in three of the 13 regions in Burkina Faso (Sud-Ouest [South-West], Centre-Sud [Central-South], and Centre-Est [Central-East]) with the highest rates of malaria transmission. One district was purposefully selected from each of the three regions, based upon its malaria epidemiology, low IPTp coverage, and the presence of active CHWs. The selected districts were Batié, Pô, and Ouargaye. In each district, four health facilities were purposefully chosen based on estimated number of pregnant women and IPTp2 coverage, for a total of 12 health facilities. These facilities served 59 villages in their catchment areas. Each village in the identified health catchment areas had at least two CHWs with at least one trained on malaria case management.

At baseline, interviews with staff and CHWs established the extent to which they were involved in malaria in pregnancy services. Three providers per facility were selected for interviews including the head of the health facility, one ANC provider, and one CHW supervisor. One CHW from each village was interviewed for the baseline survey with priority given to female CHWs. Interviews were constructed in a semi-structured format with discrete answers (yes, no; number of years) or short open-ended phrases that were entered in a spreadsheet for easy sorting and analysis.

At endline, interviews focused on the experiences of facility HCWs and CHWs in the intervention area with emphasis on uptake of service delivery tasks by CHWs. Interviews assessed their ability to execute assigned roles and responsibilities in order to identify key facilitators and requirements for further implementation of the c-IPTp programme in Burkina Faso (Table 1). Facility HCWs included in the interviews were the officer in charge, the person responsible for CHW supervision, and the person in charge of ANC services. For the endline interview, all HCWs present the day of the interview were included. Endline interviews were conducted at the time of a routine monthly clinic CHW supervisory meeting and included a mix of CHWs attending from the surrounding catchment area villages. The individuals interviewed at endline were not necessarily the same individuals interviewed at baseline. Throughout the programme roll-out study staff also observed programme execution.

**Table 1:** Enhanced responsibilities of facility healthcare workers (HCWs) and community health workers (CHWs) supporting delivery of community based IPTp in Burkina Faso

Role	Activity	Supervised by
Facility healthcare worker (HCW)	Distribute intermittent preventive treatment of malaria in pregnancy Complete and maintain study tools and documents Hold monthly meetings with CHWs to collect information on progress and discuss any challenges Provide supportive supervision of CHWs through review of collected data Provide tools for community health workers Manage sulfadoxine–pyrimethamine stock Produce reports regarding IPTp uptake	District coordinator
Community health worker (CHW)	Conduct sensitization sessions (individual and group) Refer pregnant women to healthcare facility Distribute intermittent preventive treatment of malaria in pregnancy at the community level Complete and maintain study tools and materials to document key areas such as numbers of sensitization sessions, homes visited, women referred to ANC, and doses of SP Provide completed tools to healthcare facility Manage sulfadoxine–pyrimethamine stock Produce reports regarding IPTp uptake	Facility healthcare workers

The interviews were conducted in French by trained interviewers from both the MOH and Jhpiego. To be eligible for study participation and to provide informed consent, individuals had to be able to complete a consent form. A small number of female CHWs interviewed were not literate and thus used fingerprint to confirm informed consent. Transcript data from HCWs and CHWs interviews were coded and analysed using qualitative content analysis to identify key themes and illustrative quotes. For consistency, one person led the analysis. Codes were constructed a priori using the interview guide and analysed under major themes and sub themes using standard precepts of grounded theory [21, 22]. At endline, ATLAS.ti8 Mac was used to manage sorting of main categories. Summary statements were developed for each theme along with representative quotes.

Ethical approval for this study was obtained from the Johns Hopkins Bloomberg School of Public Health Institutional Review Board (IRB) and the local IRB in Burkina Faso. US Centers for Disease Control and Prevention (CDC) staff were determined not to be engaged in human subjects research by the Office of the Associate Director for Science at the Center for Global Health.

## Results

Information about number of interviews is summarized in Table 2 with key characteristics of those interviewed at baseline in Table 3.

**Table 2:** Number of community healthcare workers (CHWs) and facility healthcare workers (HCWs) interviewed at baseline and endline

	Baseline	Endline
CHW, Female	42	19
CHW, Male	62	10
Total	104	29
Facility HCW, Female	12	6
Facility HCW, Male	23	15
Total	35	21
Total interviews	139	50

**Table 3:** Characteristics of community healthcare workers (CHWs) and facility healthcare workers (HCWs) interviewed at baseline

Facility healthcare workers	
Years of healthcare experience	4.6
Years providing malaria in pregnancy (MIP) services	4.3
Number of pregnant women seen each week	30

Community healthcare workers	
Years of experience as CHW	5.2
Number of pregnant women seen each week	4

### Baseline interviews

A total of 35 baseline facility HCW interviews were conducted. Views of facility HCWs provided in the semi-structured baseline interviews focused on the frequency of ANC contacts at the health centre level, and their awareness of CHWs and their activities at the village levels. They were asked about the information and tools used by CHWs to implement activities and the possibility of CHWs providing IPTp. All facility HCWs noted that the c-IPTp approach was a new concept and some expressed reservations about the ability of CHWs to carry out this approach *“They must be well trained to know what doses should be given… that it is not given to anyone but pregnant women.”* Others further emphasized that the approach could be feasible if CHWs were trained and monitored regularly, and community members were well informed about activity implementation, *“They need to be trained in the effects of malaria on pregnancy, on timing of administration and on filling out collection tools.”* HCWs revealed that to date few CHWs had been equipped with the medicines, educational aids and other tools needed to deliver IPTp and facilitate linkages to ANC services, noting that, *“We’re waiting for them to get their tools.”*

A total of 104 CHW interviews were conducted at baseline. CHWs had a positive view towards implementation of the community health programme with one noting there is, *“Good collaboration between the people and us.”* CHWs had been working in this role for an average of 5.2 years and referred or engaged with an average of four pregnant women each week; 60% were male. Being from the communities themselves, they felt that they were well-positioned to improve community health outcomes. However, they recognized that CHWs were not always available due to their personal responsibilities and travel, *“Sometimes it is difficult due to lack of transportation.”* All CHWs believed that they would be able to administer SP to pregnant women if they were trained by HCWs. One CHW noted that their supportive activities could reduce the workload of facility HCWs. Some female CHWs felt that female CHWs should be available to communities because some clients may be ashamed to speak to a male CHW *“Being a man and talking about something that is related to sex in the village is hard,”* or a husband could complain if he saw a man interacting with his wife. The majority of male CHWs thought that a CHW, whether male or female, could do the job if the person was well-trained, and the community accepted them.

### Endline interviews

Endline interviews were conducted with 29 CHWs and 21 facility HCWs; approximately 2/3 of those interviewed were male. Key themes and subthemes were identified within the domains explored in the interview guide (Table 4).

**Table 4:** Endline facility healthcare workers (HCW) and community healthcare workers (CHW) interviews: themes and sub-themes

Theme	Sub-theme
1. Community engagement in community based IPTp delivery	
2. Facilitators and barriers to ANC linkages	Facilitators to ANC linkages
	Barriers to ANC linkages
3. Facility healthcare worker (HCW) perceptions of CHW effectiveness	
4. Integration between CHWs and facility HCWs	Meetings with ANC providers
	Ease of CHW restocking IPTp-SP
	Incorporation of c-IPTp data collection tools
5. Gender, CHW roles and perceived effectiveness	Difference if male
	Difference if female

#### Community engagement in community based IPTp delivery

Both CHWs and facility HCWs confirmed that most communities were engaged with the c-IPTp programme. Likewise, facility HCWs and CHWs reported receiving positive feedback from the community. Community members were also said to be involved with community awareness activities, *“The community supports me in the mobilization of the population when there are community awareness sessions or mass campaigns”* and “*helps by getting villagers together for health talks.”* Other examples of support mentioned by CHWs included community members helping with clinic referrals by accompanying the pregnant woman to the clinic. Most CHWs mentioned that community members also aided in reminding women of appointments, *“The community helps us pass along the dates when we will visit households.”* Very few, only two CHWs (of 29) and two facility HCWs (of 21) said the community had not helped with the programme, though they did not note the reasons for lack of community engagement.

#### Facilitators and barriers to ANC linkages

One of the main tasks of CHWs during the project was to create awareness about the importance of ANC services and IPTp and facilitate linkages as sensitization is a key facilitator to ANC uptake. One CHW explained that, *“It is good awareness that can convince and encourage a pregnant woman to go for first ANC. It's really the sensitization that is at the root of everything in the woman at the village. If she understands… it works.”* CHWs indicated that they conducted follow-up with pregnant women at their home to encourage them to go to ANC for their own health and that of their fetus. “*The CHW must follow up with the woman to encourage her to respect her appointments. That’s what can encourage it (*ANC attendance)*”* CHWs also reported that women were aware of specific benefits such as the fact that appointments and services were free, and that they were given medications, supplements and bed nets which were motivating factors.

CHWs noted serving in a referral role to healthcare facilities. *“They see that when pregnant women are referred to the health centre by the CHW, the care would be better.”* As a result of encouragement, CHWs note that women sought them out to get a referral slip. According to CHWs, if the pregnant woman had a health problem or complication, she was likely to seek support from a CHW for many needs. *“Some women think we can treat everything. When they come, we give a referral.”*

Few CHWs (7 of 29) noted that they did not know of anything that discouraged a woman from attending ANC*.* This was partly attributed to CHW efforts which one described as follows: “*With the CHW work, women now understand. There is nothing that can discourage them from starting or continuing ANC. She starts late if she does not know she is pregnant.”* Potential reasons for discouragement mentioned by CHWs included lack of information on the importance of attending ANC, as noted by one CHW: *“Lack of knowledge of ANC can discourage pregnant women from going to the first ANC.”* Other factors included cultural traditions, mistrust of CHWs, shame to discuss the pregnancy (especially if with a male provider) or not wanting people to know she is pregnant. Additional discouraging factors include lack of family support, poor quality of care at ANC, and accessibility of the health facility. One HCW explained, *“Poor reception by health workers can lead a woman not to continue ANC at the health centre.”*

#### Facility healthcare worker (HCW) perceptions of CHW effectiveness

Overall, facility HCWs who provided ANC felt that the work of CHWs in the intervention was positive for the health of their communities. Facility HCWs served as trainers and supervisors, establishing a system that fostered contact between women and CHWs at the village level. One facility HCW said, *“From the beginning of the study we put pregnant women in contact with CHWs. When they come for ANC after getting IPTp1, we explain to them that the CHW will come to their home to give them the other doses. At the end of each month, the CHWs are informed of pregnant women in their village who should receive SP. This is how they contact them and provide SP at home.”* A facility HCW explained, *“CHWs are very useful for our work. With CHWs, ANC contacts have increased and women respect appointments. We hope that continues. CHWs are good but need to be monitored and supervised.”* The facility HCWs found that overall, CHWs had a good command of the job tools available to them, but there were difficulties for certain CHWs who could not read or write.

#### Integration between CHW and facility HCWs

CHWs perceived that their work improved the linkage between clinic and community. As one noted, *“CHWs make communication between the population and health workers easier.”* The CHWs were aware of mechanisms for them to be in contact with HCWs and specifically mentioned the standing monthly meetings. A CHW said: *“When I go to the health facility, we go over what I have done during the month and I get more SP. The health workers visit me to see what I’m doing. They come each month. After they have seen what I do, they give me work advice.”* CHWs may go to the health facility more frequently if they need support. As one CHW confirmed, “*It’s once a month that we meet with health workers to look at the registry, the stock card, report and take SP.*”

Maintaining supplies at the community level was essential for the CHW effort to function. CHWs noted no major issues of SP restocking which they did at the monthly meeting with ANC providers. As one CHW explained, “*I obtain the drug at the CSPS (health facility) with health workers. There has never been a problem getting the drug*.” If they ran out during the month, they would visit the health facility to replenish. The only possible difficulty noted was the lack of means of transport. Field supervisors confirmed that throughout the implementation there were no stockouts of SP in the three study districts.

Regarding the c-IPTp activity records (registers, monthly summary sheets), some CHWs found that the use of records was easy while others found it difficult. One CHW said: *“I use referral sheets, a monthly report, a stock inventory sheet, a register, and there is also a bag and a flip chart. The referral sheet is the easiest tool to use. The register is the most difficult.”* The study team observed CHWs using their job aids and flip charts to educate/advise pregnant women, giving the correct doses, and keeping accurate records in their villages.

#### Gender and CHW roles and effectiveness

Some facility HCWs found that either a man or a woman was able to perform the work of a CHW. They justified this by the fact that all CHWs receive the same training and tasks are not differentiated by gender in the official CHW documents from the Ministry of Health. Many confirmed the roles were the same between males and females.

Other facility HCWs found there are a few situations where gender of the CHW might make a difference. A female facility HCW observed that male CHWs could have greater influence on male villagers. *“The tasks are no different for men than for women. Men share information more often about their work as a CHW. They carry more weight when doing sensitization with other men.”* Some female CHWs reported that men were able to move more freely than women as they do not need permission to leave the house. *“It’s really easier for a man to do the job because of his freedom and his decision-making in front of other men.”* On the other hand, interacting with females in the community makes the CHW role more difficult for men. One male HCW noted it was harder for the man to approach a woman, and husbands may be upset. “*The community often finds it difficult to accept that a male CHW visits a young pregnant woman at her home (for customary or religious reasons). It is often difficult for them (husbands) to entrust their wives to a man*.”

Being a female CHW makes it easier to approach women in the community and talk to them about sensitive subjects such as pregnancy and family planning. *“A female CHW can communicate better with women. She goes to the same places (wells, market, *etc*.) and that facilitates sharing information,*” noted a male CHW. *We find that between us women, we understand each other better and can say certain things that we cannot say to a man,”* said a female CHW. Some perceived that women are less free than men and have more family constraints, including needing permission from her husband, than men which makes their work as a CHW harder. *“The female CHW can do her job well if she gets along with her husband. Her job can be difficult if she does not get along with her husband because she will not be available often.”* a male CHW remarked*.* Not being accepted by the community can also make her work hard, as women are not listened to as easily as men. *“It takes total trust in the female CHW by the community to be accepted.”* a male CHW noted.

## Discussion

IPTp is a life-saving intervention that can reduce morbidity and mortality among pregnant women and their infants. Despite long-standing recommendations, IPTp coverage has lagged, leading to efforts to explore more efficient delivery models such as community-based delivery [14, 15]. This intervention in Burkina Faso, as described in Gutman et al. was found to increase IPTp, to not reduce ANC visits [20] as had occurred in several other studies delivering c-IPTp [23, 24], and to be a safe and acceptable delivery model both from the perspectives of CHWs and facility HCWs.

At baseline, most CHWs felt capable of adding new tasks to their work. Although a few facility HCWs were initially skeptical, their main concern about ensuring well-trained CHWs was addressed by the fact that they themselves were the CHW trainers and supervisors within their own catchment areas. Facility HCWs noted the need for ongoing supervision of CHWs. In addition to training CHWs, facility HCWs held regular meetings to review progress, solve problems, and document women seen either at ANC or in the community. A systematic review of CHWs and malaria service delivery found that across many settings, training, supervision and provision of clear guidance are important factors in ensuring quality delivery of malaria services in the community [25–27]. In another study in Burkina Faso, a CHW programme did not improve outcomes among children under 5, which the authors attributed to implementation shortcomings related to training, supervision and drug stock-outs [28]. Attention to supply chain and sufficient supplies of SP was critical and has been noted as vital to ensuring CHWs can work effectively [25]. Although there is evidence of decongestion of health facilities when malaria cases are treated in the community [29], including in Burkina Faso [30], c-IPTp does not necessarily have that same impact on facility HCW burden of care due to the need to maintain ANC visits so that women receive other ANC services.

A community-clinic partnership fostered through ongoing engagement was a foundational component of the intervention and has been shown to be critical to success [31, 32], in addition to facilitating strong referral connections [33–35]. Several CHWs observed that women sometimes expected them to be able to provide more types of services than they were able to and thus referred. They didn’t identify this as an area where women were disappointed in the CHWs necessarily but it does highlight the need for clear community understanding of CHW capacities. Strong connections to a clinic were important to avoid concerns about possible reduced ANC attendance within a c-IPTp delivery programme, which had been seen in other settings [23, 24]. CHWs were motivated by the health of their communities as has been found in other studies [36]. CHWs noted delivering sensitization messages and generally strong community engagement, which has shown to be key in delivery of IPTp and malaria services to pregnant women [23, 37, 38].

Barriers to ANC attendance noted by CHWs are similar to those identified in other studies such as lack of knowledge of the importance of ANC, lack of support from family, and concern about quality of services [39]. Other studies had noted inadequate uptake of IPTp due to concerns among health staff regarding IPTp timing, as well as stock shortages of SP, which was not found in this study [25, 40].

The CHW role in recording and reporting needs to be stressed as it was an area that some CHWs noted was challenging. CHWs can learn to keep community records on IPTp, make appointments, and easily contact pregnant women in their communities to ensure they receive the next IPTp dose, but need support [31]. Monthly meetings provided an opportunity to review and summarize CHW data at the health centre and helped ensure quality of the data collected by CHWs; the meetings enabled regular supervision when health centres lacked adequate transportation resources.

While the consensus among health staff and CHWs was that both male and female CHWs could be trained to perform and carry out the same tasks, some tasks were more comfortable for either males or females. While male CHWs were in a better position to provide communication and education about the programme specifically and health generally to other males in the village, including the husbands of the pregnant women, there were some cultural limitations to their interactions with pregnant women whose husbands may not be comfortable with a male health worker making home visits. Female CHWs were reported to have better rapport with pregnant women, but less influence on male villagers, similar to a study in Kenya which found that female CHWs were perceived to provide higher quality counseling related to maternal and newborn issues [41]. The original model of the Burkina Faso community-based health promotion effort of having one female and one male CHW in each village clearly has benefits in ensuring that the total population is reached. Pairing female and male CHWs have been shown to overcome potential gender issues [42]. Reaching the husband is important. One study in Burkina Faso affirmed that husbands, having ‘‘caused’’ a pregnancy, feel a shared responsibility for their wives’ related healthcare above what they might from illnesses [43] and another study noted that spousal support for taking IPTp was attributed to effective community sensitization, emphasizing the importance of CHW efforts [44].

One limitation of this study was that it was conducted in three limited geographical areas and may not reflect the perspectives of CHWs and facility HCWs in other localities. In addition, the focused attention on IPTp and attention to availability of commodities may not reflect standard programme conditions. Additionally, the individuals interviewed at baseline were not necessarily the same as those interviewed at endline, thus some comparisons were not possible. However, it is important to note that the intervention was designed to build upon the existing CHW job description and the broader CHW training and activities reflecting the current “standard of care” of service delivery in the country. Full sustainably of this approach would require continuous training of new CHWs, as well ongoing supervision.

Following this study, the Burkina Faso Ministry of Health committed to maintaining implementation of the intervention in the communities that participated in the pilot intervention, simplifying the c-IPTp tools used by CHWs, advocating for at least one female CHW to every one male CHW in the recruitment of CHWs for each village, and advocating to make SP available at all times so that there are no missed opportunities to provide SP to pregnant women.

IPTp is a long-standing intervention to reduce malaria morbidity and more broadly further safe motherhood initiatives [45]. One of the ongoing challenges in IPTp has been bringing the intervention to nationwide scale and sustainability [5, 31, 46]. Though engagement of CHWs is broadly endorsed by WHO for primary healthcare [47], it has not been directly incorporated into malaria-specific guidance for IPTp. An update of WHO IPTp Guidelines may be helpful to clearly indicate support for community-based delivery of IPTp to stakeholders in countries with a high-burden of malaria implementing IPTp. This study demonstrates the acceptability of c- IPTp to facility HCWs and CHWs. In order to see maximum impact, it will be important to determine ways for the intervention to occur more broadly and be sustained over time.

## Conclusions

This c-IPTp study has shown that it is feasible and acceptable for CHWs, once trained, equipped, and supervised by front-line health staff to reach pregnant women and distribute multiple doses of IPTp. Ensuring regular supplies of SP and having the involvement of both female and male CHWs were facilitating factors to success. C-IPTp can increase IPTp coverage and provide more of the WHO recommended ANC contacts. This intervention could be considered as an additional approach to complement facility-based delivery of IPTp in settings with a strong CHW presence.

## Data Availability

Data has been made available on the USAID Development Data Library (DDL; https://data.usaid.gov/) as restricted access pending approval from Burkina Faso government. Data can also be requested from Jhpiego by writing to opendatahelp@jhpiego.org under a data use agreement.
